# Research Advances and Future Perspectives of Superhydrophobic Coatings in Sports Equipment Applications

**DOI:** 10.3390/molecules30030644

**Published:** 2025-01-31

**Authors:** Guoyuan Huang, Yingqing Guo, Byungchan Lee, Hong Chen, Aqiang Mao

**Affiliations:** 1 Ya’an Key Laboratory of Sports Human Science and National Physical Fitness Promotion, College of Physical Education, Sichuan Agricultural University, Ya’an 625014, China; 14760@sicau.edu.cn; 2 China Institute of Sport Science, Beijing 100061, Chinawhalesportsone@yeah.net (A.M.); 3 Department of Sport Science, Chungwoon University, Chungcheong 32244, Republic of Korea; 4 College of Food Science, Sichuan Agricultural University, Ya’an 625014, China; chenhong945@sicau.edu.cn

**Keywords:** superhydrophobic coatings, sports equipment, water-repellent properties, self-cleaning capabilities, material applications

## Abstract

In recent years, superhydrophobic coatings have attracted much attention due to their excellent water repellency and self-cleaning properties. They have broad application prospects in improving the performance and durability of sports equipment (such as clothing, footwear, balls, and protective equipment). Recent studies have shown that these coatings can improve water repellency, reduce friction, enhance traction, and extend the service life of sports equipment by preventing water absorption and reducing dirt accumulation. Despite their potential, the practical application of superhydrophobic coatings still faces many challenges, including difficulties in coating preparation, limited long-term durability, and high production costs that prevent large-scale production. This paper begins with an analysis of the current status of superhydrophobic coatings in sports equipment, from theory to application, from the basic Young’s model to the novel Contact Line Pinning Model, analyzing the advantages and disadvantages of several methods in some aspects, focusing on the most commonly used preparation methods, including the template method, the gel–gel method, the deposition method, etc., and objectively analyzing the preparation methods to match the appropriate sports equipment applications. Despite these advances, there are still significant gaps in understanding the long-term performance of these coatings under real-world conditions. The paper concludes by identifying future research directions, with a focus on improving wear resistance, improving cost-effectiveness, and assessing the environmental impact of these materials. These insights will contribute to the continued development and application of superhydrophobic coatings in the field of sports equipment.

## 1. Introduction

In recent years, superhydrophobic materials have attracted much attention due to their excellent water repellency. These materials have a unique surface micro-nano structure that minimizes contact with water droplets, resulting in phenomena such as self-cleaning, reduced friction, and enhanced durability [1,2]. Superhydrophobicity is usually defined by a water contact angle (WCA) greater than 150°, which can be explained by various theoretical models, such as Young’s model, Wenzel’s model, and Cassie–Baxter’s model. These models take into account factors such as surface roughness and surface energy that influence the water-repellent properties of materials.

Superhydrophobic materials have broad application prospects in various fields, such as the construction, automotive, and aerospace industries [3,4,5,6]. In the field of sports equipment, the application of superhydrophobic coatings is becoming increasingly important. These materials can significantly improve the performance and durability of products such as footwear, clothing, and protective equipment [7]. By reducing water absorption and improving water repellency, superhydrophobic coatings can reduce the weight of sports equipment, improve traction on wet surfaces, and improve the overall durability of equipment in harsh environmental conditions [8]. In addition, the self-cleaning properties of superhydrophobic surfaces help to keep sports equipment clean and hygienic, reducing the need for frequent cleaning and maintenance [9].

Currently, there are various methods for preparing superhydrophobic surfaces, such as the template method, sol-gel method, coating method, and deposition method. These methods achieve superhydrophobicity by controlling the surface roughness and chemical composition. For example, the template method uses a substrate with a specific morphology to adjust the reaction conditions to prepare superhydrophobic coatings with high adhesion and durability [10]. The sol-gel method synthesizes superhydrophobic coatings through the transformation of a liquid sol to a solid gel. This method can be mass-produced, and the coating properties can be adjusted as needed [11]. Although hydrophobicity is better, the equipment requirements for this method are too high. Coating methods such as spraying and dipping prepare superhydrophobic coatings by directly depositing hydrophobic solutions on the surface of the substrate. This method is simple to operate and suitable for a variety of substrates [12]. It has the disadvantage of being fast but low in durability. Deposition methods such as chemical vapor deposition (CVD) and physical vapor deposition (PVD) form superhydrophobic coatings by depositing specific chemicals or metals on the substrate surface, which can precisely control the micro-nano structure of the surface [13]. However, both have advantages and disadvantages. Physical vapor deposition has low pollution and good wear resistance, while chemical vapor deposition can control the thickness. However, the cost of both is high.

However, there are still some challenges in applying superhydrophobic materials to sports equipment. Factors such as the durability of superhydrophobic coatings under real-world conditions, production costs, and the complexity of integrating them into existing manufacturing processes may limit their widespread use in industry [14,15,16]. To improve the durability and cost-effectiveness of superhydrophobic coatings, researchers have developed a variety of methods, including new synthesis techniques [17]. For example, superhydrophobic surfaces were fabricated on copper substrates to form surfaces with pinecone-like graded micro-nano structures, and the surfaces were made superhydrophobic by assembling low-energy fluorinated components [18], which can achieve high water contact angles and low sliding angles. However, the process is complicated, and its effectiveness needs to be evaluated. These developments have brought superhydrophobic materials closer to practical applications in the sports industry, such as improving the flight stability of ball games and enhancing the water repellency and ice resistance of sports equipment [19,20].

Nevertheless, further research is required to improve the high-performance application of superhydrophobic materials in sports equipment. This paper aims to provide a comprehensive review of the current State-of-the-Art superhydrophobic coatings for sports equipment. It discusses the theoretical basis of superhydrophobicity, analyses the commonly used preparation methods, evaluates their advantages and limitations, and discusses the challenges and prospects of their application in sports equipment. By identifying the main challenges and opportunities in this field, this paper will provide guidance for future research efforts and promote the development of high-performance, durable, and affordable superhydrophobic materials in the sports industry.

The development of durable and affordable superhydrophobic coatings for sporting goods has implications beyond the sporting sector. The knowledge gained from researching and optimizing these materials for the sporting industry can be applied to other sectors, such as healthcare and food processing [21,22]. Furthermore, advancements in superhydrophobic coating technology can help develop environmentally friendly and sustainable solutions that reduce the need for frequent washing and cleaning of sporting goods, conserving water and energy [23]. The economic impact of superhydrophobic coatings in the sporting industry should not be overlooked. The successful integration of superhydrophobic coatings into sports equipment not only improves the performance and durability of the products but also provides manufacturers with a competitive advantage [24]. However, in order to fully realize the potential of superhydrophobic coatings in sports equipment and other fields, the challenges associated with their commercialization and mass production must be addressed [25]. This requires collaboration between academia, industry, and government agencies to promote innovation, technology transfer, and the widespread adoption of superhydrophobic coatings in various applications.

In this paper, the application of superhydrophobic coatings in sports equipment is systematically discussed, from the theoretical basis to the preparation methods and then to the practical applications and challenges. Through a comprehensive review of the field, it is intended to provide valuable insights for researchers, materials scientists, and industry professionals to promote research and innovation in advanced superhydrophobic materials, not only for the sports industry but also for other fields that can take advantage of the unique properties of these coatings. As the demand for high-performance, environmentally-friendly solutions continues to grow, the insights provided in this article will be invaluable in guiding future research efforts and driving the successful commercialization of superhydrophobic coatings for sporting goods and beyond.

## 2. Basic Concepts of Superhydrophobic Theory and Related Models

The phenomenon of wetting, or infiltration, occurs when a liquid interacts with a solid surface. The adhesive layer of the liquid extends across the solid’s surface and invades the solid, replacing the initial solid–gas and liquid–gas interfaces with a new solid–liquid interface. The emergence of the wetting phenomenon is typically associated with the characteristics of both the liquid and the solid [26]. With the progressive advancement in technology and a deeper comprehension of surface phenomena, various models have been developed and refined to elucidate the wetting characteristics on solid surfaces, including Young’s model, Wenzel’s model, Cassie–Baxter’s model, and Cassie–Wenzel’s model [27].

### 2.1. Young’s Model

**Figure 1:**
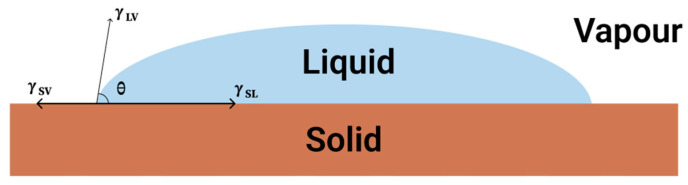
Young’s Model.

Young’s model serves as a fundamental theoretical framework for depicting the wetting behavior of liquids on solid surfaces [28]. It establishes a relationship between the contact angle of a droplet on a surface and the interfacial tensions at the three-phase contact line, which encompasses the solid, liquid, and vapor phases. According to Young’s equation, the contact angle (*θ*) can be expressed as in Equation (1) below:

(1) $$\gamma_{L}\cos\theta =\gamma_{S}-\gamma_{SL}$$

where γ represents the interfacial tensions between solid–vapor (S), solid–liquid (SL), and liquid–vapor (L) interfaces [29]. This relationship is essential for comprehending the wetting characteristics of superhydrophobic materials. The model is detailed in Figure 1. The significance of surface energy and texture is underscored in the context of superhydrophobic surfaces. The synergistic effect of low surface energy materials combined with micro- or nano-scale surface roughness results in a pronounced increase in the contact angle, thus facilitating the attainment of superhydrophobic characteristics [30]. Recent investigations have revealed that alterations to the surface architecture can further augment these superhydrophobic traits through the manipulation of both roughness and chemical attributes. For example, the incorporation of hierarchical structures can entrap air beneath water droplets, effectively minimizing the contact area and thereby enhancing the surface’s self-cleaning capabilities [31,32]. A comprehensive understanding of Young’s model and its ramifications for superhydrophobic surfaces is imperative for researchers and engineers focused on the development of advanced materials with customized wetting properties, particularly for specialized applications such as sports equipment and beyond.

### 2.2. Wenzel’s Model

**Figure 2:**
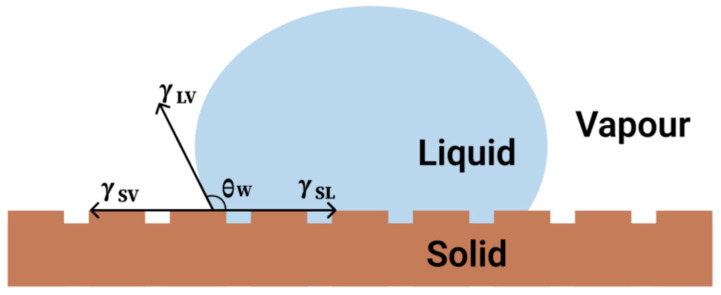
Wenzel’s Model.

The Wenzel model is a foundational theory in surface wetting that elucidates the interaction between liquids and solid surfaces, especially those that are rough or textured [33]. This model posits that the wetting characteristics of a liquid droplet on a rough surface can be defined by the contact angle, which is determined by the surface roughness and the material’s intrinsic wettability. The model is detailed in Figure 2. The Wenzel equation, which correlates the apparent contact angle with the intrinsic contact angle and the roughness factor, serves as an essential tool for elucidating the wetting dynamics on various surfaces. Recent research has underscored the necessity of integrating volume correction factors into the Wenzel model to attain a more precise depiction of liquid behavior on rough surfaces. For instance, a study by Zhang et al. introduced a volume-corrected Wenzel’s model to reconcile discrepancies noted in experimental findings [34]. Additionally, the Wenzel model is frequently compared to the Cassie–Baxter model, which describes an alternative wetting state wherein the liquid droplet rests upon the peaks of a rough surface, resulting in an elevated apparent contact angle. Studies have indicated that the Wenzel state can manifest under specific circumstances, particularly when the liquid infiltrates the surface roughness, a phenomenon that was rigorously explained by Chen et al. [35]. The transition between the Cassie and Wenzel states is critical for applications involving superhydrophobic materials, as the management of the wetting state can profoundly influence the efficacy of surfaces engineered for water repellency or self-cleaning properties.

The Wenzel model provides a more detailed perspective on wettability by exploring the influence of surface roughness on the contact angle between a solid and a liquid. The model quantifies the ratio of the actual contact area to the projected area of a solid surface by introducing a roughness factor *r*, where *r* > 1 indicates a rough surface. The Wenzl equation is given by Equation (2) below:

(2) $$\cos\theta_{w}=\frac{r\left(\gamma_{SV}-\gamma_{SL}\right)}{\gamma_{LV}}$$

In this equation, *θ_w_* is the contact angle of a liquid on a rough surface, and *γ_SV_, γ_SL_*_,_ and *γ_LV_* represent the interfacial tensions of the solid–vapor, solid–liquid, and liquid–vapor interfaces, respectively. Using this relationship, the Wenzel model reveals how surface roughness can enhance wettability or non-wettability by increasing the actual contact area. This model is particularly useful for superhydrophobic materials with high roughness, as it helps us understand and predict how roughening can significantly increase the contact angle of liquids, thereby achieving better superhydrophobic properties.

In addition to the foundational concepts of the Wenzel model, recent progress has concentrated on the characterization of intermediate wetting states on nano/microstructured surfaces. These investigations have disclosed that the presence of microstructures can give rise to intricate wetting behaviors that neither the Wenzel nor Cassie–Baxter models can fully account for [36]. This has stimulated the formulation of more advanced models, such as the Bi-Gaussian stratified wetting model, which considers the multi-scale roughness of surfaces and provides a more thorough comprehension of liquid behavior on these intricate substrates [37]. Furthermore, the implications of the Wenzel model extend beyond mere wetting phenomena; they significantly contribute to various applications, including the design of environmentally friendly superhydrophobic materials. Research has shown that optimizing surface roughness alongside chemical modifications can enhance the superhydrophobicity of surfaces, leading to superior performance in environmental and energy-related applications [38]. Additionally, advancements in nanostructured surfaces have facilitated the characterization of condensation phenomena, which are critical. In summary, the Wenzel model is a fundamental aspect of wetting theory, offering crucial insights into the interaction between liquids and textured surfaces. Current research efforts are continually enhancing our comprehension of this model and its various applications, facilitating the development of innovative materials and technologies that utilize surface-wetting principles. The investigation into novel surface architectures and their effects on wetting behavior is expected to significantly advance the creation of sophisticated materials with customized properties suitable for a multitude of applications.

### 2.3. Cassie–Baxter’s Model

**Figure 3:**
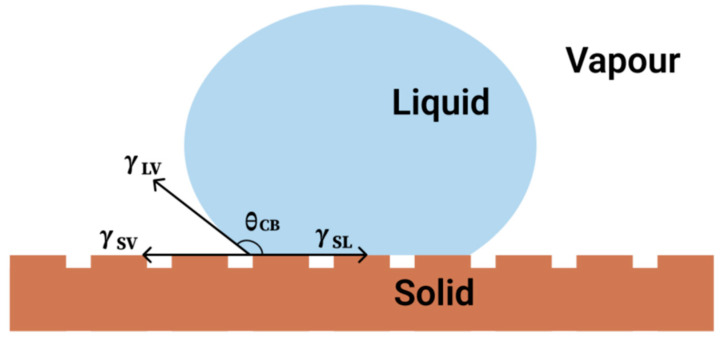
Cassie–Baxter’s Model.

The Cassie–Baxter model serves as a crucial theoretical framework for elucidating the wetting behavior of liquids on textured surfaces. The model is detailed in Figure 3. According to this model, when a liquid droplet rests on a rough surface, it does not completely infiltrate the recesses of the texture; rather, it remains situated on the peaks of the rough features [39]. This phenomenon results in the formation of air pockets beneath the droplet, consequently leading to a pronounced increase in the apparent contact angle relative to that of a smooth surface. The Cassie–Baxter equation, which connects the contact angle of a droplet on a textured surface to the contact angles observed on a flat surface and the proportion of the solid–liquid interface, is vital for predicting the wettability of diverse materials [40]. Recent investigations have corroborated the relevance of the Cassie–Baxter model across various surfaces, including those exhibiting hierarchical textures produced through advanced manufacturing techniques such as laser ablation and micro-injection molding [41].

The Cassie–Baxter model provides a high-level framework for understanding and designing superhydrophobic surfaces, which takes into account the partial wetting of liquids on rough or textured surfaces. The model assumes that the contact point of a droplet on a solid surface coexists with a composite interface of solid and gas, resulting in a more complex three-phase contact line. According to the Cassie–Baxter equation, the contact angle *θ_CB_* in Equation (3) can be expressed as follows:

(3) $$\cos\theta_{CB}=f_{SL}\cos\theta_{0}-f_{LV}\cos\theta_{1}$$

where *f_SL_* and *f_LV_* represent the area fractions of the solid–liquid and liquid–gas interfaces, respectively; *θ*_0_ is the liquid’s natural contact angle on a smooth solid surface, and *θ*_1_ is the liquid’s contact angle on a pure gas interface. This analysis helps us understand how droplets on microstructured surfaces achieve super-high contact angles by reducing the actual liquid–solid contact area.

The Cassie–Baxter model highlights the importance of surface texture and chemical composition, which are crucial to achieving and maintaining superhydrophobicity. By precisely adjusting the microstructure and chemical properties of a surface, its hydrophobic properties can be greatly enhanced. For example, by increasing the surface roughness on a micrometer or nanometer scale, the proportion of gas can be effectively increased, thereby increasing the contact angle of the drop and reducing adhesion by reducing direct contact between the liquid and the solid.

Moreover, the Cassie–Baxter model has been examined in relation to biocompatible surfaces and superhydrophobic coatings, augmenting our understanding of how surface textures affect liquid behavior. For example, fractal theory has been employed to create flexible superhydrophobic surfaces, illustrating that microstructural design can effectively modulate wetting characteristics. Additionally, this model has implications in a myriad of fields, encompassing self-cleaning surfaces, anti-fogging technologies, and biomedical applications aimed at developing surfaces that reduce bacterial adhesion. The interactions between the Cassie–Baxter and Wenzel regimes, where droplet behavior shifts in accordance with surface properties, have also been extensively studied, emphasizing the intricate nature of wetting phenomena.

Furthermore, the Cassie–Baxter model has proven to be essential in estimating hydrophobic surface structures, enabling researchers to anticipate droplet behavior on complex geometries [42]. This aspect is particularly pertinent for the design of surfaces designated for specific applications, such as microdroplet manipulation in lab-on-a-chip devices, where controlled wetting is imperative. The ongoing investigation of the Cassie–Baxter model continues to unveil new insights into the dynamics of wetting and dewetting processes, especially under varying environmental conditions, such as during freezing and melting cycles. Overall, the Cassie–Baxter model remains a fundamental concept in surface science, bridging theoretical insights with practical applications in material design and engineering.

### 2.4. Rolling Angle and Contact Angle Hysteresis Theory

The rolling angle and contact angle hysteresis are pivotal parameters for comprehending the wettability and surface characteristics of superhydrophobic materials. The rolling angle denotes the slope at which a droplet begins to roll off a surface, while contact angle hysteresis represents the disparity between the advancing and receding contact angles of a droplet on a given surface. This hysteresis reflects the energy barrier that must be surmounted for the droplet to transition from a stable state to a rolling state and then to a dynamic state. High contact angle hysteresis is typically regarded as undesirable in superhydrophobic surfaces, as it can result in water retention and diminish self-cleaning efficiency [43]. Theoretical frameworks, such as the Cassie–Baxter and Wenzel models, elucidate the influence of surface roughness and chemical composition on these contact angles. In the context of superhydrophobic surfaces, the ideal scenario encompasses a low rolling angle paired with minimal hysteresis to guarantee optimal droplet mobility and self-cleaning characteristics, which are crucial for a variety of applications, including textiles and coatings.

### 2.5. Contact Line Pinning Model

The contact line pinning model is a key theoretical framework for understanding and describing the wetting behavior of droplets on microstructured or chemically heterogeneous surfaces. A study has re-examined this phenomenon using the minimum potential energy principle and proposed a new model based on triple contact line (TCL) pinning [44]. Contact line pinning refers to the phenomenon in which the contact line of a droplet is pinned at a specific location on the surface due to a discontinuity in the local geometric or chemical properties, thereby affecting the shape and motion of the droplet. This phenomenon has important application value in the fields of wetting science and surface engineering, especially when designing superhydrophobic surfaces and regulating the behavior of liquids on surfaces.

Contact angle hysteresis is an important parameter for evaluating the influence of contact line pinning, and it represents the difference between the advancing contact angle and the receding contact angle of a droplet moving over a surface. This hysteresis reflects the energy barrier that a droplet must overcome to transition from a stable to a dynamic state. In the design of superhydrophobic surfaces, it is generally desirable to have as small a contact angle hysteresis as possible, as this helps to reduce the adhesion of water droplets to the surface and thus improves the self-cleaning properties of the surface.

In the context of the contact line pinning model, an ideal superhydrophobic surface should have a low rolling angle and minimal contact angle hysteresis. The rolling angle indicates the tilt at which a droplet begins to roll off a surface. A low rolling angle means that a droplet can roll easily at a very small tilt, which is a key characteristic for achieving an efficient self-cleaning surface. For example, in textile and coating applications, this characteristic ensures that liquids are quickly removed from the surface, reducing the build-up of contaminants.

### 2.6. Summary of Superhydrophobic Theory

In order to systematically compare the characteristics and application scope of various theoretical models of superhydrophobicity, we have conducted a comprehensive analysis of the main theoretical models, the results of which are summarised in Table 1. As shown in Table 1, the Young model is mainly applicable to the analysis of ideal smooth surfaces, while the Wenzel model and Cassie-Baxter model focus more on the influence of surface roughness. The rolling and contact angle hysteresis (RACAH) and contact line pinning model (CLPM) further extend the scope of application, taking into account not only surface roughness and chemical heterogeneity, but also droplet dynamics and the design of superhydrophobic surfaces. This systematic comparison helps us to better understand and choose the theoretical model that is suitable for a particular application scenario.

### 2.7. Influence of Temperature on the Superhydrophobic Model

When designing sports equipment, it is important to consider the effect of temperature on the performance of superhydrophobic materials. Superhydrophobic surfaces effectively repel water through their microstructure and chemistry, but changes in temperature can affect these properties. For example, high temperatures may cause deformation of the surface microstructure, reducing its water droplet repellency and thus reducing the superhydrophobicity of the material [45]. In addition, changes in temperature may also affect the chemical stability of the surface coating, which in turn changes its hydrophilic and hydrophobic characteristics. In the design of outdoor sports equipment, such as skis, windsurfing boards, and camping equipment, the equipment is often exposed to extreme temperature conditions, from hot summers to cold winters. This requires superhydrophobic surfaces to maintain their performance at different temperatures to ensure that the surface of the equipment remains dry, improving comfort and performance stability. Recent studies have shown that smooth superhydrophobic surfaces with appropriate nanoscale roughness exhibit superior water repellency at low temperatures when supercooled drops impinge at high speeds [46]. Furthermore, further research has revealed that water droplets on hierarchical superhydrophobic surfaces exhibit evaporation characteristics that do not match predictions from conventional models under heating conditions, emphasizing the important role of evaporative cooling in determining the evaporation behavior of droplets [47]. Therefore, when developing and testing superhydrophobic sports equipment, the temperature factor must be fully considered to ensure that the material maintains its excellent hydrophobic properties even under temperature fluctuations, thereby extending the service life of the equipment and improving its overall performance.

## 3. Classification and Methods for Preparing Superhydrophobic Coatings

Naturally occurring hydrophobic substances typically display remarkable hydrophobic traits, serving as templates for the development of hydrophobic coatings. These coatings are designed to replicate the water-repellent properties observed in natural entities such as lotus leaves and specific insect wings, enabling them to effectively repel water, remain dry, and thereby prevent contamination and corrosion [48]. Presently, the predominant techniques for fabricating superhydrophobic coatings encompass the template method, sol-gel technique, spraying method, vapor deposition, chemical etching, and a range of innovative approaches.

### 3.1. Template Method

**Figure 4:**
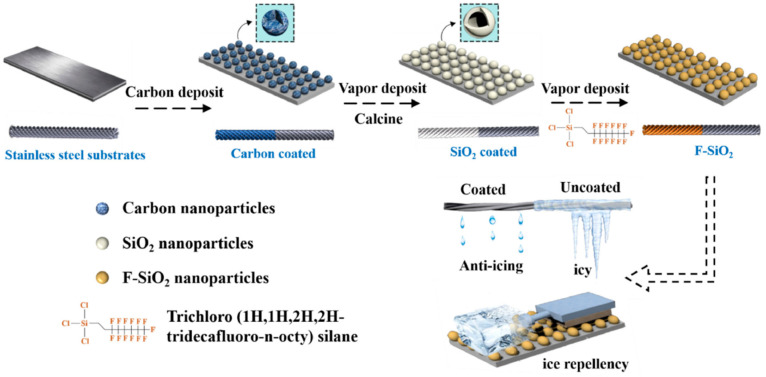
Fabrication of superhydrophobic anti-icing coatings through template and deposition methods [49].

The template approach entails the utilization of a substrate possessing a defined morphology to regulate reaction conditions for the fabrication of superhydrophobic coatings. Coatings produced through the template method exhibit high adhesion strength to the substrate, remarkable durability, and extended stability. However, this technique necessitates stringent criteria for template selection and preparation, which may render it impractical for certain substrates and elevate preparation costs as well as complexity. Consequently, the template method is predominantly employed for the production of large-area substrates. For instance, Cao et al. [49] initially created a soot layer on the stainless-steel substrate’s surface, subsequently positioning the template substrate in an evaporating dish, and executed chemical vapor deposition for 24 h using tetraethyl orthosilicate (TEOS) and ammonia water. Following this, they employed trichloro (1H, 1H, 2H, 2H-perfluorooctyl) silane vapor deposition to develop a superhydrophobic anti-icing F-SiO_2_ coating on the metallic surface, as illustrated in Figure 4. The dashed arrows in the figure indicate the sequential steps in the coating preparation process, leading to the final anti-icing performance test.

**Figure 5:**
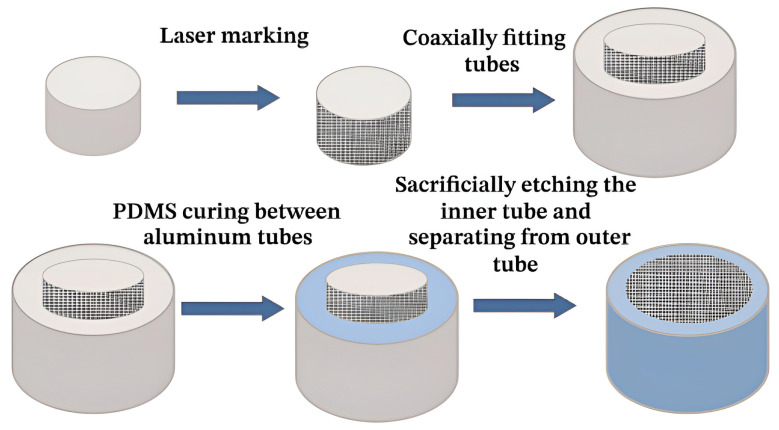
Preparation process of superhydrophobic flexible tubes [50].

Furthermore, Wang et al. [50] utilized prepared 6061 aluminum alloy tubes as templates, employing polydimethylsiloxane (PDMS) as replicas. By manipulating the laser etching distance on the aluminum alloy tube surfaces, they successfully produced superhydrophobic hoses exhibiting both water and blood repellency, with the fabrication process detailed in Figure 5.

The template method has significant potential for the fabrication of superhydrophobic coatings on sportswear and footwear. By using templates with specific surface morphologies, it is possible to create micro-nano-structured surfaces that exhibit excellent water repellency and durability. For example, using a template with a hierarchical structure resembling the surface of a lotus leaf can result in a superhydrophobic coating that mimics the self-cleaning properties of the natural template. These coatings can be applied to various sportswear and footwear materials, such as fabrics, polymers, and composites, to improve their performance in wet or humid conditions. The high adhesion strength and durability of template-derived superhydrophobic coatings make them suitable for applications in sportswear and footwear that are subject to frequent use and wear, such as running shoes, hiking boots, and athletic clothing.

### 3.2. Sol-Gel Method

**Figure 6:**
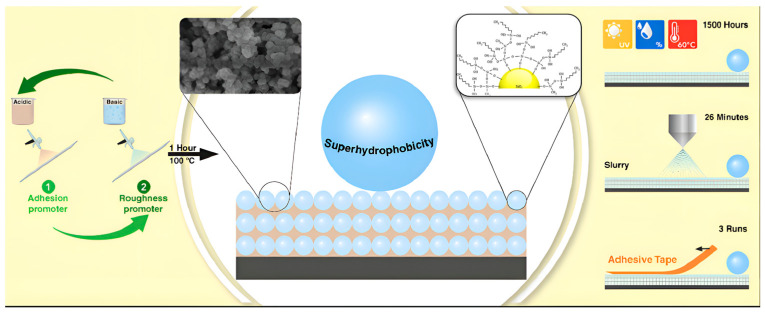
Schematic diagram of the preparation, mechanism, and testing of superhydrophobic coatings on glass using the sol-gel process [53].

The sol-gel method is a flexible technique for synthesizing superhydrophobic coatings through the transition from a liquid sol to a solid gel phase [51]. This methodology facilitates the integration of diverse functional groups and nanoparticles, thereby enhancing the hydrophobicity of the resultant product [52]. Materials generated via the sol-gel process can establish a network structure, which improves the mechanical stability and longevity of the coatings. This method is particularly advantageous for large-scale production and can be tailored to yield coatings with varying properties, making it applicable in industries such as textiles and construction materials. Hashjin et al. [53] along with Duan et al. [54], Li et al. [55], and Ke et al. [56] modified the conventional sol-gel process by employing tetraethyl orthosilicate (TEOS) and low surface energy compounds as precursors, in conjunction with nano-silica (SiO_2_) to create micro-nano structures. By adjusting parameters such as pH, reaction duration, temperature, and the amount of reactants employed, they successfully enhanced the properties of the coatings. In specific proportions, superhydrophobic coatings were successfully synthesized, exhibiting a contact angle exceeding 150°, as illustrated in Figure 6. The green circular arrows in the figure represent the sol-gel transition process, while the black arrow indicates the preparation sequence of the superhydrophobic coating.

**Figure 7:**
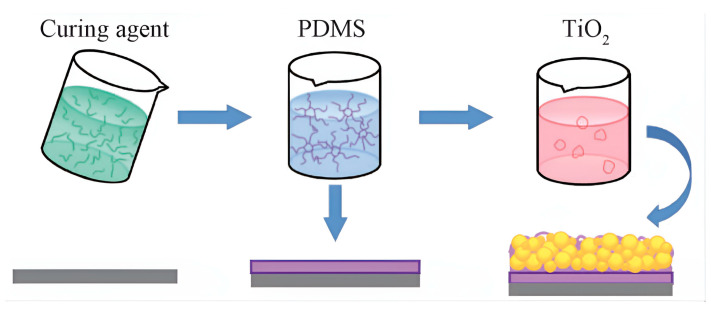
Preparation process of superhydrophobic flexible tubes [33].

Xue Xinyu et al. [57] enhanced TiO_2_ through the incorporation of sodium laurate (C_12_H_23_NaO_2_) and sodium hexametaphosphate ((NaPO_3_)_6_), subsequently blending it with the high-viscosity elastomer polydimethylsiloxane (PDMS). This approach facilitated the development of durable superhydrophobic TiO_2_/PDMS coatings on carbon steel surfaces, with the fabrication process detailed in Figure 7. In the bottom panel, the three horizontal planes represent the bare substrate surface, the PDMS-coated surface, and the final TiO_2_-deposited surface with superhydrophobic properties, respectively. The arrows indicate the coating and deposition processes between these surfaces.

The sol-gel method offers a versatile approach for the preparation of superhydrophobic coatings on sportswear and footwear. By incorporating various functional groups and nanoparticles into the sol-gel matrix, it is possible to create coatings with tailored hydrophobicity and mechanical properties. For instance, the addition of fluorinated silanes or silica nanoparticles can significantly enhance the water repellency and durability of the coatings. These superhydrophobic coatings can be applied to a wide range of sportswear and footwear materials, such as textiles, polymers, and leather, to improve their performance in wet conditions. The sol-gel method allows for the fabrication of coatings with varying thicknesses and morphologies, which can be optimized for specific applications in sportswear and footwear. For example, thinner coatings can be used on fabrics to maintain their flexibility and breathability, while thicker coatings can be applied to shoe soles for enhanced durability. The sol-gel process also enables the large-scale production of superhydrophobic coatings, making it suitable for the manufacturing of sportswear and footwear with improved water resistance and self-cleaning properties.

### 3.3. Coating Method

**Figure 8:**
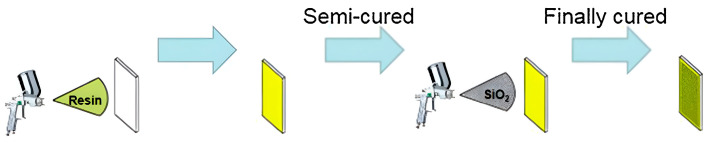
Preparation of a wear-resistant, superhydrophobic SiO_2_/silicone-modified polyurethane composite coating through a two-step spraying method [63].

Techniques such as spraying or dip-coating serve as direct methodologies for the application of superhydrophobic materials to various surfaces [58]. These processes involve the deposition of a hydrophobic solution onto a substrate, which adheres to form a thin hydrophobic film. The selection of the hydrophobic agent, in conjunction with the application method, substantially influences the coating’s efficacy [59]. These techniques boast significant adaptability and are applicable to a broad range of substrates, including fabrics and metals, thus rendering them appealing for commercial utilization. The advantages of these methods include their simplicity, rapid application, widespread applicability, and robust operational capabilities. However, they are not without drawbacks; challenges such as stringent coating requirements during spraying, inconsistent thickness, and the instability of the resultant superhydrophobic coatings persist [60]. Presently, a primary obstacle in the preparation of superhydrophobic coatings via spraying lies in the challenge of preserving the coating’s integrity while facilitating swift preparation [61]. Liu et al. [62] employed four non-fluorinated modifiers—dodecyltrimethoxysilane (DTMS), hexadecyltrimethoxysilane (HTMS), octadecyltrimethoxysilane (OTMS), and polydimethylsiloxane (PDMS)—to modify EP-SiO_2_ and fabricate stable superhydrophobic coatings on glass substrates. Details regarding the preparation process, coating wettability, self-cleaning capabilities, and mechanical stability are depicted in Figure 8. The light blue arrows in Figure 8 indicate the sequence of the curing process, showing the progression from initial coating to a semi-cured state and finally to a fully cured coating. Guo et al. [63] utilized silica gel to modify polyurethane surfaces, applying it onto pretreated glass slides with a spray gun. After removing the semi-cured polyurethane, they sprayed SiO_2_ dispersion, which was then dried to yield fully cured superhydrophobic SiO_2_/silicone-modified polyurethane (SiO_2_/SiPU) coatings. Zhang et al. [64] integrated carbon nanofibers (CNF) with a high aspect ratio into fluorosilicone resin (FSR) to create a uniform network structure. By combining this with carnauba wax (CW) and modified fluoro rubber (MFR), they achieved a superhydrophobic FSR/CNF/CW/MFR coating exhibiting a water contact angle of 157°.

The coating method, particularly spraying and dip-coating techniques, has significant potential for the application of superhydrophobic coatings on sportswear and footwear. These methods allow for the rapid and uniform deposition of hydrophobic materials onto various substrates, making them suitable for large-scale production. For example, spraying techniques can be used to apply superhydrophobic coatings onto fabrics used in sportswear, such as jerseys, shorts, and socks, to improve their water resistance and moisture management properties. Dip-coating methods can be employed to create superhydrophobic surfaces on shoe soles and uppers, enhancing their durability and self-cleaning capabilities. The coating method also enables the incorporation of various hydrophobic agents, such as silicones, fluoropolymers, and waxes, which can be selected based on the specific requirements of the sportswear and footwear application. Additionally, the thickness and morphology of the coatings can be controlled by adjusting the coating parameters, such as the concentration of the hydrophobic solution, the spraying distance, and the dipping time. This flexibility allows for the optimization of the superhydrophobic properties and mechanical stability of the coatings for different sportswear and footwear materials and designs.

### 3.4. Deposition Method

In contrast to the technique of coating hydrophobic particles, the deposition method provides another effective way to obtain superhydrophobic surfaces. In deposition techniques such as chemical vapor deposition (CVD) and physical vapor deposition (PVD), superhydrophobic coatings can be formed by depositing specific chemicals or metals on the surface of the substrate. These methods allow precise control of micro- and nanostructural features on the surface, thereby effectively constructing the desired rough texture. Chemical vapor deposition uses chemical reactions to generate superhydrophobic coatings on the surface of the substrate. By controlling the reaction conditions, such as temperature and pressure, the thickness and structure of the coating can be finely adjusted [65]. Physical vapor deposition forms a coating by physically evaporating the target material and condensing it on the surface of the substrate. PVD technology can be used not only to deposit metals and oxides but also a variety of materials, such as plastics and fibers, to improve their superhydrophobic properties [66].

#### 3.4.1. Physical Vapor Deposition

**Figure 9:**
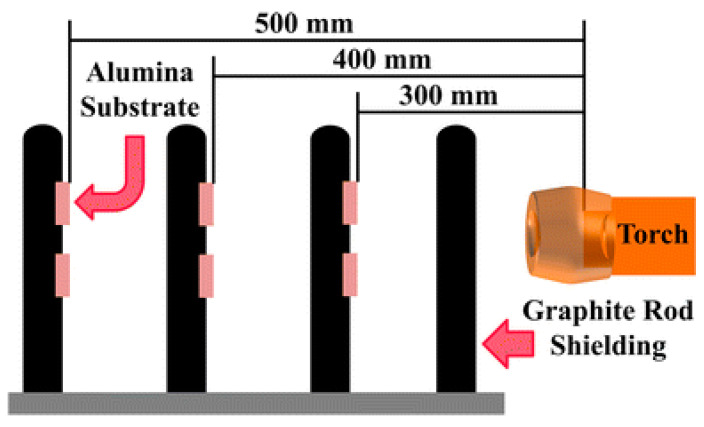
A super-hydrophobic surface prepared by lanthanide oxide ceramic deposition through the PS-PVD process [68].

Physical Vapor Deposition (PVD) involves the transformation of a material from a solid to a vapor state in a vacuum environment and then condensing it on a substrate to form a thin film. This technology covers a variety of different deposition methods, including cathodic arc deposition, electron beam physical vapor deposition, evaporation deposition, sputtering, and ion plating. The common feature of these methods is that they can form thin films with extremely high adhesion on the substrate. PVD technology is widely used in many fields, such as aerospace, automotive manufacturing, medical devices, cutting tools, and textiles, because of its high efficiency and the ability to impart special properties to materials. In the textile industry, PVD technology is becoming an important innovation tool. By changing the surface properties of textiles, new development opportunities have been brought to textile design and functional textiles [67]. Li et al. [68] prepared a ceramic superhydrophobic surface using the PS-PVD process, as shown in Figure 9. Voznesenskaya et al. [69] deposited a carbon nanostructured coating on a polyurethane substrate using the PVD method.

Yu et al. [70] prepared TiAlSiN coatings with different silicon contents using PVD, systematically studied their chemical composition, microstructure, and mechanical properties, and found that an appropriate silicon content can significantly improve the microhardness and adhesion of the coating, as well as improve the performance of milling hardened steel. Wang et al. [71] characterized the coatings using PVD micro-scratch tests and friction and wear tests on TiN coatings prepared on a GCr15 substrate. The results showed that the coatings prepared using the composite method had a smoother surface and better overall properties, such as high cohesion and hardness, resulting in a lower wear rate.

Physical vapor deposition (PVD) methods have great potential for the manufacture of superhydrophobic coatings on sportswear and footwear. PVD techniques (such as sputtering, evaporation, and cathodic arc deposition) enable precise control of the composition, thickness, and morphology of the coating, making them suitable for the manufacture of micro- and nanostructured surfaces with excellent water repellency and durability. For example, PVD can be used to deposit thin films of hydrophobic materials (such as fluoropolymers or silicones) on a variety of sportswear and footwear substrates, including textiles, polymers, and leather. These coatings can significantly improve the water repellency, self-cleaning properties, and abrasion resistance of sportswear and footwear, thereby enhancing their performance and lifespan in wet environments. In addition, PVD methods can also be used to create multi-layer coatings with gradient hydrophobicity, thereby further optimizing the wetting properties and mechanical stability of the surface. PVD coatings have high adhesion and uniform coverage and are therefore suitable for sportswear and footwear that are frequently used and worn, such as running shoes, hiking boots, and sportswear.

#### 3.4.2. Chemical Vapor Deposition

**Figure 10:**
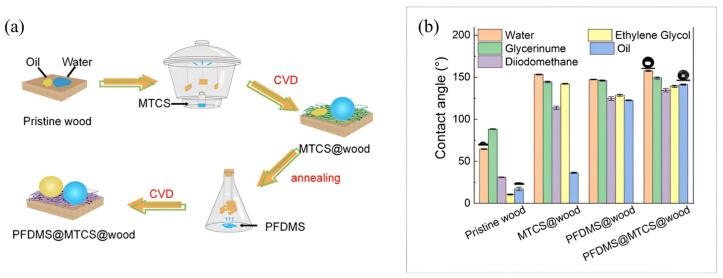
Process and contact angle testing of superhydrophobic coatings prepared by vapor deposition method [73].

Chemical Vapor Deposition (CVD) constitutes a technique utilized for the formation of superhydrophobic coatings through the deposition of specific chemicals or chemical groups onto the substrate surface, leading to the generation of a micro-nano-structured thin film under controlled deposition time and temperature [72]. While this methodology is relatively straightforward and user-friendly, it still faces challenges related to the regulation of coating thickness and environmental safety during the experimental procedure. Jian et al. [73] implemented a two-step CVD process, applying methyltrichlorosilane (MTCS) and 1H,1H,2H,2H-perfluorodecyltrimethoxysilane (PFDMS) to birch wood surfaces to produce superhydrophobic coatings. The preparation process and the static contact angles of various droplet sizes on the wood are illustrated in Figure 10a,b, where bar graphs show the contact angles for different test liquids (Glycerinume, Oil, and Diidomethane) under various treatment conditions. The black circles (○) in Figure 10b indicate specific measured values: pristine wood showed a water contact angle (WCA) of 64.6° and oil contact angle (OCA) of 17.2°, while after MTCS treatment, the WCA increased to 153.3°. The PFDMS@MTCS@wood treatment achieved both superhydrophobicity and oleophobicity with a WCA of 157.7°.

**Figure 11:**
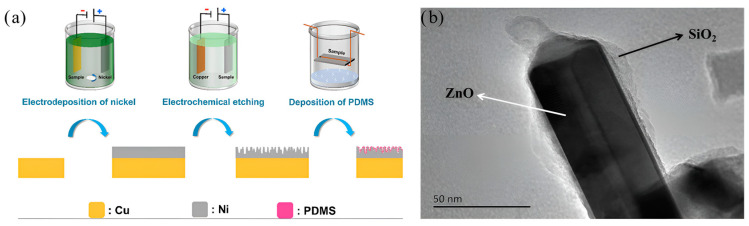
(**a**): Process and mechanism of preparing superhydrophobic coatings by vapor deposition method [74]. (**b**): UV-durable superhydrophobic ZnO/SiO_2_ nanorod arrays on an aluminum substrate using catalyst-free chemical vapor deposition and their corrosion performance [75].

Ye et al. [74] combined electrochemical etching with CVD technology to integrate electroplated nickel with the PDMS hydrophobic material, as shown in Figure 11a, to provide a double layer of protection for the copper substrate, thereby improving its suitability for practical applications. Zhang et al. [75] directly synthesized ZnO nanorod arrays on aluminum substrates, subsequently coating them with SiO_2_ films via pulsed laser deposition, as demonstrated in Figure 11b. After modification, the Al/ZnO/SiO_2_ nanorod arrays exhibited superhydrophobic characteristics.

### 3.5. Etching Method

In comparison to coating techniques, where hydrophobic particles are applied, etching methods offer alternative approaches for achieving superhydrophobic surfaces. The formation of superhydrophobic coatings on substrate surfaces can be achieved through the application or deposition of particles [76]. Techniques such as laser etching and chemical etching facilitate the development of superhydrophobic surfaces by either physically etching or selectively corroding the substrate through physical or chemical means. These methods serve to construct a specific rough texture on the surface or to lower the activation energy. Given that the adhesion between the coating and the substrate does not need to be addressed, these approaches provide significant benefits, including enhanced stability, robust corrosion resistance, and prolonged service life [77].

#### 3.5.1. Chemical Etching Method

Chemical etching entails the immersion of glass or metal components into an etching solution. At ambient temperature or elevated temperatures, the components designated for etching dissolve, thereby achieving the desired etched design and a specific surface roughness that fosters the formation of a superhydrophobic surface [78]. In comparison to laser etching, chemical etching offers advantages such as reduced costs and the ability to manage substrate parameters, shapes, and orientations. Conversely, its limitations include inconsistent uniformity and ineffectiveness for substrates that pose challenges for etching [79].

To mitigate the negative impact of etching on the strength of substrates, Zhang et al. [80] developed an innovative superhydrophobic glass surface employing fluorocarbon resin combined with SiO_2_ particles. When the nanoparticle concentration was 25%, and the immersion duration was 30 min, the surface achieved a contact angle of 159.5° and a rolling angle of 1.0°. Remarkably, by immersing the substrate in a diluent (butyl acetate), the surface demonstrated capabilities for self-healing against mechanical damage. Kumar et al. [81] successfully produced a superhydrophobic surface on an aluminum plate utilizing a blend of concentrated nitric and hydrochloric acids, achieving a contact angle of 162°. This superhydrophobic coating preserved its hydrophobic characteristics for 15 h in an acidic environment with a pH of 5 and exhibited stability for up to 4 days in an alkaline solution with a pH of 11. Notably, the employment of highly concentrated strong acids or bases in the preparation of superhydrophobic surfaces results in significant pollution, underscoring the pressing need for the development of eco-friendly techniques for superhydrophobic surface fabrication.

Wang et al. [82] adeptly created adjustable patterned copper oxide micro-bump surfaces through a combination of wet chemical oxidation, photolithography, and electron beam evaporation techniques. These micro-bumps were formed from well-aligned copper oxide nanowhiskers, featuring noticeable cavities in the elevated regions (a result of photoresist removal during oxidation). Following treatment with fluoroalkylsilane, the multi-level structured surfaces exhibited exceptional superhydrophobicity, characterized by high contact angles exceeding 160° and notable water condensation performance. The microscale dimensions of the multi-level surfaces significantly influence the nucleation, growth, and detachment of droplets. When the diameter of the micro-bumps was approximately 15 μm, and the cavity diameter was around 1.5 μm, condensation droplets would spontaneously release from the surface. Such superhydrophobic surfaces present considerable potential in applications related to self-cleaning, anti-fogging, water collection, and heat transfer [83].

Chemical etching methods have shown great promise in the fabrication of superhydrophobic surfaces for sports equipment. By selectively etching metal substrates, such as aluminum or titanium alloys, it is possible to create micro-nano hierarchical structures that enhance the water repellency and self-cleaning properties of the surface. These superhydrophobic surfaces can be applied to various sports equipment, including bicycle frames, golf clubs, and ski poles, to improve their performance in wet or humid environments. Additionally, chemical etching can be used to create superhydrophobic patterns on glass or plastic surfaces, such as goggles or helmets, to prevent fogging and improve visibility during sports activities. The chemical etching process can be optimized to achieve the desired surface roughness and morphology while minimizing the impact on the mechanical properties of the substrate. Furthermore, the incorporation of environmentally friendly etching solutions and post-treatment methods can enhance the sustainability and practicality of superhydrophobic surfaces prepared by chemical etching for sports applications.

#### 3.5.2. Laser Etching Method

**Figure 12:**
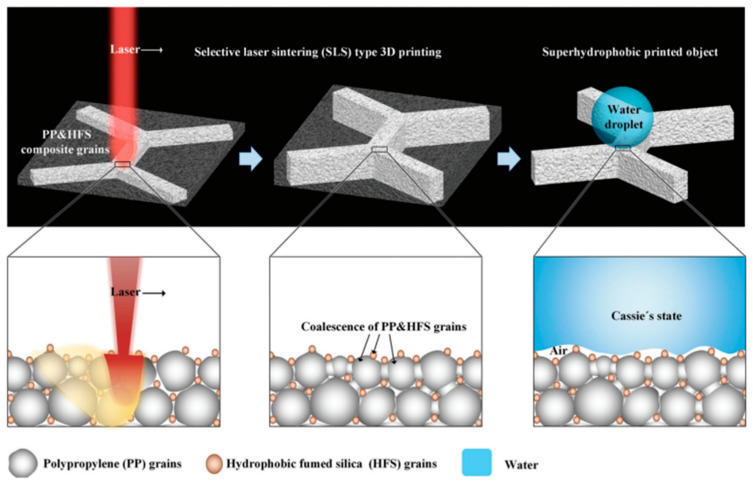
SLS-type 3D printing strategy [56].

The laser etching technique employs high-energy laser ablation on the substrate surface to initiate a series of reactions via photoelectric or photothermal effects, thereby modifying the surface roughness and achieving a superhydrophobic surface. This method boasts advantages such as single-step processing, rapid operation, and absence of pollution; however, it is generally associated with higher costs [84]. Song et al. [85] employed femtosecond laser ablation in conjunction with pentafluorooctanoic acid immersion to engineer an aluminum surface with modifiable adhesion characteristics. By varying the laser parameters, the adhesion characteristics of ace were found to vary significantly. Jing et al. [86] utilized a femtosecond laser system to manipulate grid-like microstructures on a glass substrate. As the pulse energy was increased, micro-nano particles began to accumulate on the glass surface. Following this, a chemical modification of the surface was performed using low-surface-energy fluorinated alkylsilane compounds, resulting in a contact angle measurement of 152.7°. Notably, the superhydrophobic glass surface demonstrated sustained hydrophobic properties even after undergoing six abrasion tests. These findings suggest that the micro-nano structures induced by the laser are heavily influenced by the energy input and play a critical role in adhesion characteristics. To expedite the laser ablation process and minimize energy consumption, Wu et al. [87] adopted selective laser sintering (SLS) 3D printing technology, incorporating hydrophobic vapor-phase SiO_2_ particles as the hydrophobic agent. The concurrent sintering of the polymer provided sufficient mechanical integrity, yielding a low-adhesion superhydrophobic surface with a desired three-dimensional configuration (refer to Figure 12). This fabricated structure maintained its anti-wetting properties after undergoing various wear tests, including knife cuts, three sandpaper abrasion tests, tape tests, and flowing sand impact tests, thereby demonstrating remarkable mechanical stability.

In an effort to improve economic efficiency and reduce the duration of laser ablation, Lin et al. [88] implemented an ultrafast laser multi-processing technique to generate a nanoscale rough surface. They subsequently applied a coating of fluorinated alkylsilane molecules to enhance hydrophobicity. By subjecting the etched glass to heat in a dry environment for one hour, the surface was swiftly modified through chemical deposition, leading to a significant decrease in preparation time. The resultant glass coating exhibited a contact angle of 161.2° ± 0.4° and a rolling angle of 2° ± 1°. It retained excellent superhydrophobic properties even after exposure to high pressure, water immersion for 168 h, and water flow impact for 0.5 h, with thermal stability reaching up to 500 °C and a remarkable visible light transmittance of 92%. This methodology is characterized by stability, structural controllability, and suitability for large-scale applications, thus providing substantial benefits in the fabrication of superhydrophobic surfaces on glass substrates.

Laser etching technology is promising for preparing superhydrophobic surfaces on various sports equipment materials. By using high-energy laser pulses to form micro- and nanostructures on metal, polymer, or glass substrates, excellent water repellency and self-cleaning properties can be obtained. For example, the surface of a golf ball can be coated with a laser-etched superhydrophobic coating to reduce the adhesion of water and dirt, thereby improving its aerodynamics and durability. Similarly, laser-etched superhydrophobic patterns on sports goggle lenses prevent fogging and improve visibility in wet or rainy conditions.

### 3.6. Plasma Technology

**Figure 13:**
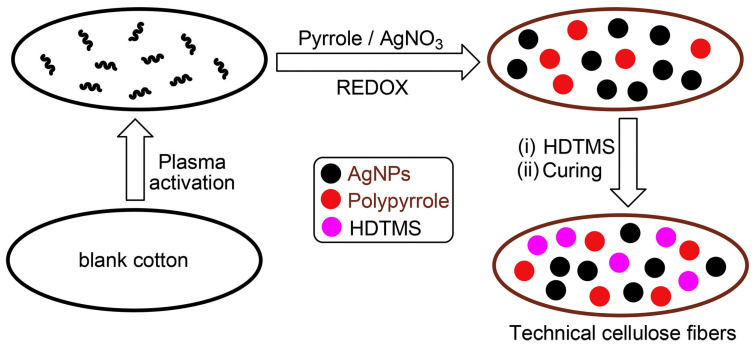
Plasma treatment toward electrically conductive and superhydrophobic cotton fibers by in situ preparation of polypyrrole and silver nanoparticles [89].

Plasma material preparation is an advanced material synthesis and modification technology that uses the unique physical and chemical properties of plasma to introduce specific structures and functions on the surface of materials or in the bulk phase. Plasma is a quasi-neutral ionized gas composed of free electrons, ions, neutral atoms, and molecules. By applying energy such as an electric field, magnetic field, or electromagnetic wave, the gas can be ionized to form a plasma. In material preparation processes, plasma can provide high-energy particles, excited particles, and chemically active particles, which can interact with materials in complex ways to achieve functions such as material synthesis, deposition, etching, and surface modification. Katouah et al. [89] plasma-treated cotton fibers, as shown in Figure 13. This treatment forms a hydrophobic surface, significantly increasing the water contact angle on the cotton fiber surface, thereby giving the cotton fiber excellent superhydrophobic properties and providing a viable solution for improving the water repellency of sportswear. Xu et al. [90] used the dip plasma crosslinking technique to prepare a poly(dimethylsiloxane)-graft-poly(ethylene terephthalate) (PDMS-g-PET) fabric. This technique does not change the wearing comfort of the fabric, which is very suitable for sportswear. Thanks to the wrinkled structure of the PDMS film, this superhydrophobic fabric has excellent durability, can resist repeated washing and physical wear, and maintains a high water contact angle, making it suitable for a variety of outdoor and sportswear.

Xu et al. [91] showed that cotton and PET fabrics treated with argon-assisted capacitively coupled plasma (CCP) technology exhibited excellent hydrophobicity, and in particular, cotton coated with LMA (Cotton-g-LMA) showed better water repellency and mechanical durability in sportswear applications. This technology improves the performance of sportswear, ensuring durability and comfort during frequent washing and high-intensity activities. It is ideal for outdoor and sportswear.

Plasma technology shows great potential for the preparation of superhydrophobic surfaces on sports equipment and clothing. By subjecting various substrates (such as fabrics, polymers, or metals) to plasma treatment, their surface chemistry and morphology can be altered, thereby enhancing their water repellency and self-cleaning properties. For example, plasma polymerization can be used to deposit a thin film of a hydrophobic polymer (such as PDMS or fluoropolymers) on the surface of sportswear fabrics to form a durable and breathable superhydrophobic coating. This can significantly improve the water repellency and moisture management properties of the garment, ensuring wearer comfort and performance in wet environments. Similarly, plasma etching can be used to create micro- and nano-roughness on the surface of metal or polymer substrates used in sporting equipment such as bicycle frames or helmets, thereby enhancing their water repellency and reducing the adhesion of dirt and debris. The versatility and scalability of plasma technology make it an ideal method for the mass production of superhydrophobic surfaces for sporting goods. Moreover, the environmentally friendly and energy-saving nature of the plasma process is in line with the growing demand for sustainable high-performance sports equipment and clothing.

### 3.7. Summary of Superhydrophobic Coating Preparation Methods

Various methods have been developed for preparing superhydrophobic coatings, each with its distinct advantages and limitations. As summarized in Table 2, these methods include template method, sol-gel method, coating method, physical and chemical vapor deposition, chemical etching, laser etching, and plasma technology. The template method offers durability and mass production capabilities but involves complex processes and high costs. The sol-gel method provides strong hydrophobicity but requires stringent physical and chemical conditions. While coating methods feature fast speed and wide applicability, they suffer from low stability and durability. Physical and chemical vapor deposition methods, along with etching techniques and plasma technology, each present unique advantages in terms of process control and coating quality, though they face challenges related to cost, environmental impact, and parameter optimization. The selection of materials, primarily including silane, polymers, nanoparticles, metal oxides, and fluorine compounds, plays a crucial role in determining the final coating properties.

## 4. Applications of Superhydrophobic Materials in Sportswear and Footwear

The integration of superhydrophobic coatings in sports apparel and footwear has been achieved through various methods, such as template-assisted synthesis, sol-gel processing, coating techniques, chemical vapor deposition, physical vapor deposition, chemical etching, laser etching, and plasma technology. These methods have enabled the development of high-performance superhydrophobic coatings that effectively inhibit moisture penetration, ensuring that the garments and footwear remain dry, which is particularly advantageous for outdoor sports and adverse weather conditions [92]. For instance, sol-gel processing and coating techniques have been widely used to create superhydrophobic coatings on fabrics, providing excellent waterproof and breathable properties. The coatings facilitate the escape of sweat and water vapor, thereby keeping the wearer dry and comfortable during physical exertion [93]. Additionally, superhydrophobic coatings prepared by chemical vapor deposition and plasma technology hinder the adhesion of dirt and dust to surfaces, allowing for easy cleaning with a simple rinse. This capability significantly decreases the frequency of washing, thereby prolonging the lifespan of the apparel [94]. By minimizing the intrusion of moisture and contaminants, the durability of these products is enhanced. Although the presence of superhydrophobic coatings is currently limited to certain high-end outdoor sports apparel and professional athletic footwear, their adoption is steadily increasing. With ongoing technological advancements and decreasing costs, it is anticipated that these coatings will be more widely utilized in sports equipment in the foreseeable future [95].

### 4.1. Waterproof Breathability and Its Regulation Principles

**Figure 14:**
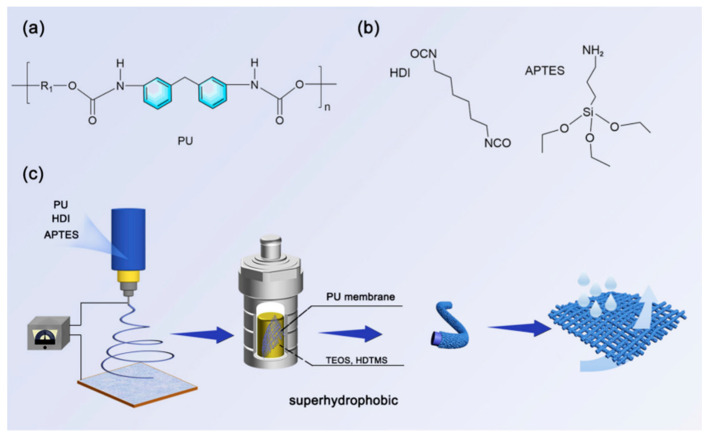
Chemical structures of (**a**) PU and (**b**) HDI, APTES. (**c**) Preparation of PU/SiO_2_ waterproof, breathable, and infrared-invisible nanofibrous membranes (schematic) [99].

Superhydrophobic materials are essential in improving the waterproof and breathable attributes of sportswear and footwear. These materials repel liquid water while allowing moisture vapor to escape, thus ensuring comfort during physical activities. The fundamental principle hinges on micro- and nano-structured surfaces that entrap air, creating a barrier against liquid water while facilitating the passage of water vapor. Dual functionality is imperative for athletes who necessitate equipment capable of performing optimally across diverse environmental conditions [96]. Superhydrophobic surfaces typically utilize specialized micro- and nano-structures that markedly decrease the contact area and adhesion of water, thus facilitating waterproof effects. For instance, when rain or perspiration interacts with a superhydrophobic surface, the resultant water droplets assume spherical forms and rapidly roll away, preventing the saturation of clothing and footwear. This property holds particular significance in outdoor activities such as hiking, running, and mountaineering, which often entail extended exposure to moisture-laden environments [97]. Furthermore, superhydrophobic materials not only confer waterproof advantages but also ensure commendable breathability. This is attributable to the micro- and nano-structures of these materials, which effectively obstruct the penetration of liquid water while permitting the passage of water vapor molecules [98]. Consequently, sweat generated during vigorous physical activities can evaporate swiftly, averting moisture accumulation within the clothing, thereby enhancing overall comfort and performance. Composite nanofibers were prepared using electrospinning and hydrothermal-assisted sol-gel methods. Polyurethane was modified with hexamethylene diisocyanate (HDI) and aminopropyltriethoxysilane (APTES) and then electrospun to prepare nanofiber membranes. Subsequently, the above-mentioned fiber membrane was grafted and modified with SiO_2_ to prepare a microporous membrane with excellent waterproof and breathable properties. Figure 14 shows the preparation process of the PU/SiO_2_ nanofiber membrane. The blue arrows in Figure 14c indicate the sequential preparation steps from spray coating to PU membrane formation and final superhydrophobic surface development. The key components in the process include the spray coating device, the substrate for coating deposition, the PU membrane formation setup, and the resulting superhydrophobic nanofiber membrane structure.This material has superhydrophobic and breathable properties, making it very suitable for use in outdoor sports equipment [99].

To attain this dual functionality of waterproof breathability, the design and production of superhydrophobic materials necessitate careful consideration of various factors. These encompass material selection, surface structure design, and meticulous control of manufacturing processes [100]. With advancements in contemporary technologies, such as laser etching, chemical vapor deposition, and nano-coating techniques, it has become feasible to accurately control and optimize these micro- and nano-structures, resulting in sportswear and footwear exhibiting outstanding waterproof and breathable characteristics [101].

In conclusion, the integration of superhydrophobic materials in sportswear and footwear not only elevates the functionality and comfort of these products but also significantly enhances their adaptability for diverse extreme environments. This represents a noteworthy technological progression for both recreational enthusiasts and elite athletes [102].

### 4.2. The Self-Cleaning Mechanism of Superhydrophobic Coatings

**Figure 15:**
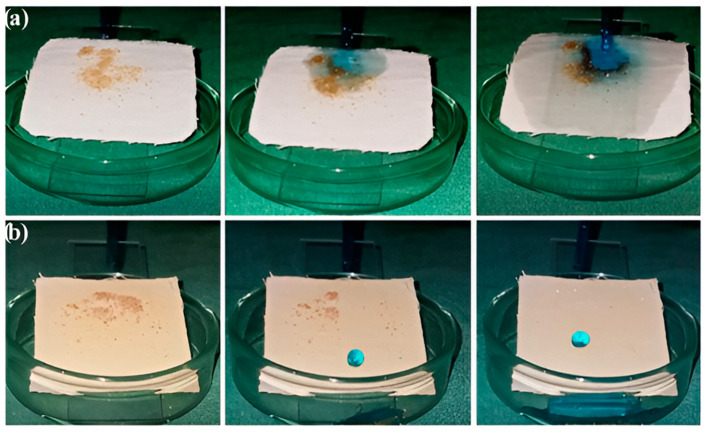
Self-cleaning characteristics of the (**a**) pristine cotton fabric and (**b**) SA/CeO_2_-cotton fabric against fine dust particles.

The self-cleaning mechanism exhibited by superhydrophobic coatings has ushered in transformative developments within modern materials science, particularly demonstrating substantial potential in the domain of sportswear and footwear applications. In the related study, dust particles were sprayed onto both the original fabric and the superhydrophobic coated fabric for measurement, as shown in Figure 15a,b. During the experiment, water was dripped onto the original and superhydrophobic coated cotton fabrics using a glass pipette. These samples were placed on a glass slide with an appropriate sliding contact angle (10°). Due to its purely hydrophilic nature, the cotton fabric absorbs the water droplets when they come into contact with the fabric surface. Dust particles accumulate on the fabric surface, as shown in Figure 15a. In contrast, the superhydrophobic coated cotton fabric, with its high surface roughness and low surface energy, exhibits dust-repellent and self-cleaning properties. When water droplets fall on the modified cotton fabric surface, dirt particles roll off along with the water droplets, cleaning the surface of the fabric (Figure 15b). The designed cotton fabric successfully demonstrated the lotus effect, indicating that garments with superhydrophobic coatings prepared by methods such as sol-gel processing and coating techniques possess certain self-cleaning properties [103].

The fundamental principle underlying this mechanism is primarily attributed to the lotus effect, a phenomenon observed in nature that elucidates the behavior of water droplets on superhydrophobic surfaces. By examining the lotus effect, we can gain profound insights into how superhydrophobic coatings facilitate self-cleaning, thereby minimizing cleaning frequency and prolonging product lifespan [104]. The lotus effect is characterized by water droplets forming nearly perfect spheres with contact angles exceeding 150 degrees on superhydrophobic surfaces, enabling them to roll off the surface [105]. This rolling action effectively dislodges surface dirt and contaminants. When water droplets make contact with a superhydrophobic coating prepared by methods such as chemical vapor deposition or plasma technology, their elevated contact angle and reduced adhesion allow them to roll off effortlessly. During this rolling process, water droplets transport dirt and particulates away from the surface, thereby achieving a self-cleaning effect [106]. This mechanism presents practical advantages, including decreased cleaning frequency, extended product longevity, and sustained performance stability. Currently, while the utilization of superhydrophobic coatings predominantly resides within the high-end market, ongoing technological advancements and cost reductions are anticipated to broaden the application of these coatings across a wider array of sports equipment in the future [107].

### 4.3. Enhancing Durability and Comfort

The incorporation of superhydrophobic coatings in sports apparel and footwear is increasingly recognized as a significant technological innovation for enhancing performance. The fundamental benefit of this coating lies in its remarkable waterproof properties. Superhydrophobic coatings prepared by various methods, such as template-assisted synthesis, sol-gel processing, and physical vapor deposition, are remarkably effective in minimizing moisture penetration and absorption, which significantly prolongs the lifespan of various materials [108]. Conventional sports equipment frequently experiences water-related damage in humid environments, resulting in deterioration or loss of shape over time. In contrast, the implementation of superhydrophobic coatings substantially alleviates this risk.

Beyond their waterproofing capabilities, superhydrophobic coatings offer exceptional resistance to stains and odors. This characteristic simplifies the maintenance of sportswear following vigorous exercise, reducing the likelihood of persistent stains and effectively controlling unpleasant odors. This aspect is particularly vital for athletes, as the cleanliness and comfort of their gear have a direct impact on their performance and psychological state during rigorous training sessions and competitions [109]. Additionally, the lightweight and flexible nature of superhydrophobic coatings prepared by methods such as chemical vapor deposition and plasma technology ensures that athletes can wear these garments without feeling encumbered, promoting a more unrestricted movement experience. Whether participating in running, workouts, or various high-intensity activities, athletes can engage confidently in diverse environmental conditions without concerns regarding discomfort or moisture. In conclusion, superhydrophobic coatings not only enhance the durability and comfort of sports apparel and footwear but also serve as a significant advantage for athletes’ performance. With continuous technological advancements, the incorporation of superhydrophobic coatings in athletic gear presents a promising avenue for future innovation, ultimately contributing to an exceptional competitive experience for athletes [110].

## 5. Applications of Superhydrophobic Materials in Sports Equipment

Superhydrophobic materials, with their outstanding water-repellent, anti-fouling, and self-cleaning properties, show great potential for application in the field of sports equipment. By applying superhydrophobic coatings to the surfaces of balls, fitness equipment, outdoor sports gear, and electronic devices, the performance and durability of these items can be significantly enhanced under various environmental conditions. Despite current challenges related to material durability and cost control, ongoing technological innovations and process optimizations are expected to enable broader applications of superhydrophobic materials in sports equipment in the future, further improving the sports experience and equipment performance.

### 5.1. Improving Flight Stability of Balls

Applying superhydrophobic coatings to the surface of sports balls significantly enhances their flight stability by reducing water adhesion under wet conditions. This treatment lowers the surface tension between the ball and water, allowing the ball to maintain excellent performance even in rainy or humid environments [111]. Specifically, the superhydrophobic coating effectively prevents the formation of water films on the ball’s surface, thereby avoiding weight increase and deformation caused by moisture [112]. As a result, the ball’s flight trajectory becomes more stable and predictable, enabling athletes to better control the direction and speed of the ball during games and training sessions [113].

In sports where ball trajectory is critically important, such as soccer and golf, the advantages of superhydrophobic coatings are particularly evident. Soccer players can continue to pass and shoot accurately in rainy conditions, ensuring the smooth progress of the game. Golfers, on the other hand, can maintain the stability of their shots on wet courses, achieving better control over the golf ball’s trajectory [114]. In summary, superhydrophobic coatings not only enhance the physical performance of the ball but also boost athletes’ confidence and improve their overall game experience.

Moreover, the application of superhydrophobic materials reduces the maintenance difficulty of balls in humid environments and extends their lifespan [115]. The promotion of this technology not only contributes to the overall quality of sports equipment but also drives technological advancements and development in the field of sports. With continuous technological innovation and application optimization, the future application prospects of superhydrophobic materials in sports equipment will be even broader, providing athletes with better game and training environments.

### 5.2. Application of Superhydrophobic Materials in Other Sports Equipment

Superhydrophobic materials, with their outstanding water-repellent, anti-fouling, and self-cleaning properties, show great potential for application in the field of sports equipment. By applying superhydrophobic coatings to the surfaces of balls, fitness equipment, outdoor sports gear, and electronic devices, the performance and durability of these items can be significantly enhanced under various environmental conditions. Despite current challenges related to material durability and cost control, ongoing technological innovations and process optimizations are expected to enable broader applications of superhydrophobic materials in sports equipment in the future, further improving the sports experience and equipment performance [116].

#### 5.2.1. Waterproofing of Fitness Equipment

The application of superhydrophobic coatings in fitness equipment is becoming increasingly popular, primarily for providing waterproofing treatment. This application is crucial for maintaining the integrity and performance of the equipment in environments with sweat and moisture [117]. By effectively repelling moisture, these coatings can prevent rust and corrosion, significantly extending the lifespan of fitness equipment and ensuring safety during use [118].

Superhydrophobic coatings can form a protective barrier on both metal and plastic surfaces of the equipment, preventing the penetration of sweat and moisture and reducing mechanical failures and wear caused by humid environments. This treatment is especially beneficial for equipment frequently used and exposed to large amounts of sweat, such as treadmills, exercise bikes, and strength training machines. These coatings protect the mechanical components and electrical systems of the equipment from moisture damage, thereby avoiding short circuits or other failures caused by humidity.

Moreover, the application of superhydrophobic coatings greatly simplifies the cleaning and maintenance of fitness equipment. As these coatings prevent stains and bacteria from accumulating on surfaces, cleaning staff can easily maintain the cleanliness and hygiene of the equipment with simple wiping. This not only raises the hygienic standards of gyms and fitness centers but also provides users with a healthier and safer workout environment.

#### 5.2.2. Enhanced Corrosion Resistance of Sports Equipment

Superhydrophobic coatings offer significant advantages in enhancing the corrosion resistance of sports equipment. Whether it is outdoor sports gear like skis and bicycles or water sports equipment such as surfboards and kayaks, these coatings provide excellent protection, allowing the equipment to maintain high performance and durability in various harsh environmental conditions [119].

Superhydrophobic coatings can form a protective barrier on the surface of sports equipment, preventing the intrusion of moisture, salt, and other corrosive substances. This is particularly important in marine environments, as saltwater is highly corrosive and can accelerate the oxidation and damage of metal components. By applying superhydrophobic technology, water sports equipment can better resist corrosion caused by saltwater, thereby extending its lifespan and reducing maintenance costs [120].

**Figure 16:**
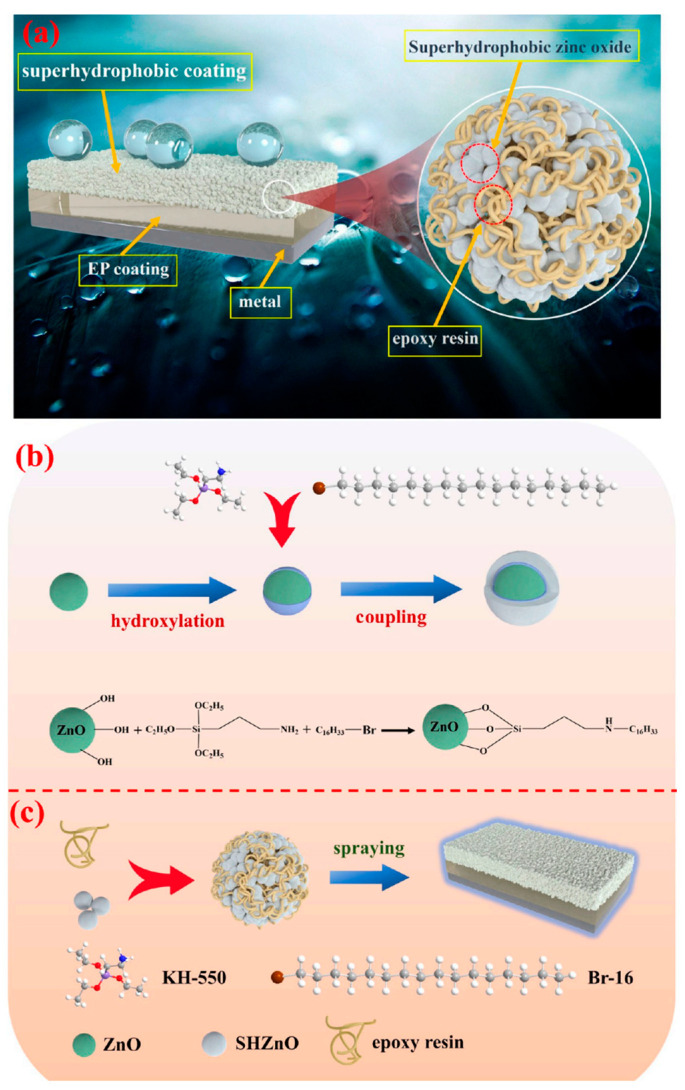
(**a**) Superhydrophobic coatings prepared using EP and modified ZnO. (**b**) Preparation of superhydrophobic zinc oxide particles. (**c**) Preparation process of superhydrophobic zinc oxide coating [121].

Superhydrophobic coatings can also effectively prevent damage to equipment in wet and muddy environments. In outdoor activities such as trail running and mountain biking, equipment is often exposed to mud and moisture, which can accelerate wear and corrosion. Superhydrophobic coatings allow water droplets and mud to quickly roll off the surface of the equipment, reducing damage caused by mud and moisture and maintaining the equipment in good working condition. In common spray methods, superhydrophobic zinc oxide particles and epoxy resin are used as binders that can bind onto the surface of the substrate. Through the curing process of epoxy resin, superhydrophobic particles are fixed on the surface of the substrate, achieving surface superhydrophobicity. As shown in Figure 16a, where the red arrow indicates the reaction/mixing process between KH-550 and other components, superhydrophobic zinc oxide particles were sprayed onto the surface of waterborne epoxy resin using a spraying method, endowing the waterborne epoxy coating with superhydrophobic properties. This significantly improves the corrosion resistance of waterborne epoxy resin. During the initial soaking period, the EIS results of the coating showed better corrosion resistance than traditional oil-based epoxy resins, with an impedance value of 1010 Ω · cm^2^. In addition, within the first 90 h of immersion, the coating exhibits excellent immersion resistance similar to that of a perfect coating. In addition, the coating also has excellent mechanical properties, self-cleaning performance, anti-icing performance, etc. This superhydrophobic coating is highly suitable for the anti-corrosion requirements of metal sports equipment [121].

Additionally, superhydrophobic coatings have self-cleaning properties, making the maintenance of sports equipment simpler and more convenient. Since dirt and microorganisms are less likely to adhere to superhydrophobic surfaces, users can easily restore the cleanliness of the equipment with a simple rinse [122]. This not only saves time and effort in cleaning but also maintains the hygiene of the equipment, enhancing the user experience.

#### 5.2.3. Enhance the Water Resistance and Ice Resistance of Sports Equipment

**Figure 17:**
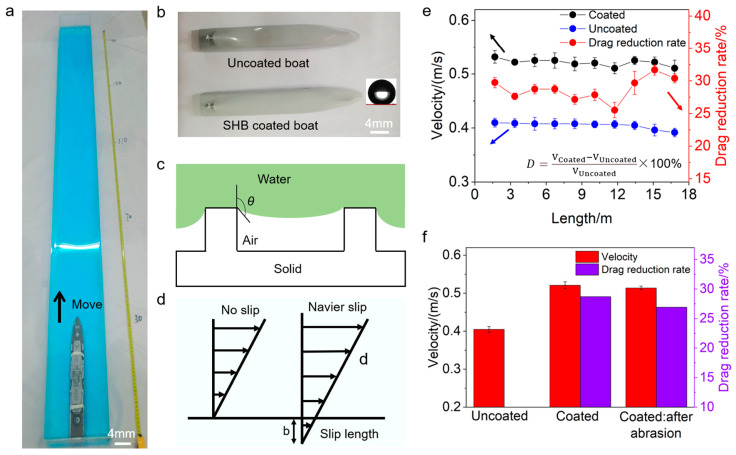
Sailing experiments on a model boat coated by prepared superhydrophobic coating [123]. (**a**) Schematic diagram of sailing experiments. (**b**) Optical images of model ships with uncoated and SHB coated boat (with contact angle measurement inset). (**c**) Microstructure of air layer. (**d**) Drag reduction mechanism of SHB coating at underwater gas-water interface. (**e**) Sailing test of the boat with and without SHB coating. (**f**) Left: moving velocity of different model boats (from left to right: Uncoated boat, boat modified with SHB coatings before abrasion and after abrasion); right: corresponding to the drag reduction rate of the SHB-coated boat before and after abrasion. The error bars denote standard deviations, obtained from three test results for each sample.

The application of superhydrophobic coatings can improve the water resistance performance of sports equipment. In a related study, the researchers conducted sailing experiments on a model ship coated with a superhydrophobic coating to evaluate its drag reduction performance (Figure 17). The experimental results showed that the superhydrophobic coating significantly increased the average sailing speed of the model ship, demonstrating its effective drag reduction capabilities (Figure 17b). The drag reduction mechanism of the superhydrophobic coating can be summarized in two main aspects: first, the coating surface changes the solid–liquid contact to solid–air contact, effectively reducing friction (Figure 17c); second, the presence of air bubbles promotes interface slip, further reducing the friction between the fluid and the ship’s surface (Figure 17d). The experimental data indicated that the average speed of the coated ship was higher than that of the uncoated ship, which confirms the notable drag reduction effect of the superhydrophobic coating [123].

In addition, the researchers conducted continuous sailing experiments (Figure 17e), and the results showed that the drag reduction effect remained almost stable as the sailing distance (16.8 m) increased, with no significant changes in the drag reduction rate, further verifying the long-lasting effect of the superhydrophobic coating. To assess the mechanical robustness of the coating, the drag reduction performance of the coated ship was tested after wear (Figure 17f). The results showed that the drag reduction rate changed minimally, from 28.7% (0.521 m/s) to 26.9% (0.514 m/s), indicating that the superhydrophobic coating has excellent mechanical durability and can provide sustained drag reduction performance over extended periods of use. Therefore, the superhydrophobic coating demonstrated good overall performance in both drag reduction and mechanical stability, making it suitable for long-term underwater applications. Therefore, superhydrophobic coatings can be applied to sports equipment such as kayaking, improving athletic performance by reducing water resistance.

In terms of anti-icing capabilities, superhydrophobic coatings also demonstrate excellent performance. When applied to ski equipment, these coatings effectively reduce the adhesion of ice and snow, thereby lowering friction and enhancing speed performance [124]. The superhydrophobic surface of ski equipment prevents ice and snow from adhering, maintaining the smoothness and flexibility of the skis, even in wet and cold environments, ensuring optimal gliding conditions. This not only increases skiing speed but also reduces the physical exertion and operational difficulty for athletes during skiing [125].

In extreme environments, the anti-icing capability of superhydrophobic coatings has a significant impact on the performance of sports equipment. For example, in sailing, applying such coatings to sails can effectively prevent icing [126]. Ice formation increases the weight of the sail, reducing its flexibility and overall performance. Superhydrophobic coatings ensure that the sail surface remains ice-free, maintaining its lightweight and efficient state. By reducing the ice and snow load, sails can still perform optimally under cold conditions, ensuring the smooth progress of competitions and the safety of athletes [127].

## 6. Challenges in Current Research and Future Development Directions

Superhydrophobic coatings, as a cutting-edge technology, possess a vast range of potential applications. Due to their remarkable water-repellent properties, these coatings demonstrate significant promise across various fields, including self-cleaning surfaces, anti-corrosion coatings, anti-icing and anti-fogging applications, and oil–water separation. In the sports arena, superhydrophobic coatings are also garnering increasing attention, with potential applications in sports equipment, outdoor sports facilities, and athletic apparel. However, actual research and applications face numerous challenges and difficulties, which limit the large-scale commercialization and widespread use of these coatings. By continuously innovating and integrating superhydrophobic coatings with sports equipment, it is possible to further expand the application areas of these coatings while also providing new directions for the innovation of sports gear.

### 6.1. Long-Term Stability, Cost, and Scalability Challenges

Firstly, the long-term stability of superhydrophobic coatings is a major issue. Environmental factors such as UV radiation, physical wear, and chemical corrosion can degrade the hydrophobic performance of the coatings. In sports applications, such as outdoor facilities like football fields, basketball courts, and running tracks, prolonged exposure to the natural environment requires the coatings to have excellent weather resistance and durability [128]. Ensuring the longevity of these coatings in practical applications remains a pressing challenge.

Secondly, production costs and scalability are significant challenges. Many advanced manufacturing techniques, such as Chemical Vapor Deposition (CVD) or specialized etching methods, can produce high-performance superhydrophobic coatings. However, these techniques are often expensive and difficult to scale up, limiting the feasibility and economic viability of superhydrophobic coatings in commercial applications [129]. In the sports sector, cost-effective manufacturing methods are particularly crucial, as large-scale sports facilities and equipment maintenance require economical solutions.

Additionally, current research needs to address the adaptability of superhydrophobic coatings in different application scenarios. In the sports field, various types of sports equipment and facilities have diverse performance requirements for the coatings. For example, skis need to be resistant to low temperatures and prevent ice and snow buildup, while swimsuits require reduced water resistance and increased durability. This necessitates higher standards for the design and manufacturing of the coatings [130].

Lastly, eco-friendliness and sustainability are also important directions for future research and development of superhydrophobic coatings. Researchers need to develop coatings that not only exhibit excellent performance but are also environmentally friendly and recyclable to meet increasingly stringent environmental regulations. For instance, coatings for sports facilities and equipment must comply with environmental standards to minimize their negative impact on the environment [131].

### 6.2. Future Research Focus Areas and Technological Prospects

Future research on superhydrophobic coatings is likely to focus on several key areas, including the development of environmentally friendly materials and processes, enhancing self-cleaning properties, and improving the mechanical durability of the coatings. Additionally, increasing attention is being paid to integrating superhydrophobic coatings with smart technologies, such as sensors and responsive systems, to create multifunctional surfaces. These advancements could lead to innovative applications across various fields, including healthcare, textiles, and consumer electronics, paving the way for a new generation of superhydrophobic coatings [132].

In recent years, the application of superhydrophobic coatings in sports equipment has achieved significant progress and accomplishments. These coatings, with their unique hydrophobic properties, have been integrated into various sports equipment, from footwear to protective gear, enhancing their performance, durability, and user comfort. The use of superhydrophobic coatings not only improves the waterproof properties of these products but also maintains their lightweight and breathable characteristics, which are crucial in high-performance sports.

However, despite these encouraging developments, it is important to recognize the existing gaps in research and application. Future research should focus on understanding the long-term durability of superhydrophobic coatings under various environmental conditions and their impact on athletic performance. Additionally, the potential ecological impacts of these coatings need to be explored, particularly the sustainability and recyclability of superhydrophobic coatings used in sports equipment.

Moreover, balancing the differing perspectives in current research is crucial. On the one hand, some studies emphasize the performance advantages of superhydrophobic coatings. On the other hand, some express concerns about their manufacturing processes and potential environmental impacts. Future research should address these discrepancies by establishing standardized testing methods and clear application guidelines, ensuring the responsible use of superhydrophobic coating technology in sports equipment.

Looking to the future, the potential for superhydrophobic coatings in the sports industry is immense. Continuous innovation can lead to the development of more advanced materials that not only enhance performance but also prioritize environmental sustainability. Researchers and manufacturers should collaborate to share knowledge and resources, maximizing the benefits of superhydrophobic coating technology while minimizing its negative impacts. By adopting an interdisciplinary approach that combines materials science, engineering, and environmental research, we can pave the way for the next generation of sports equipment that meets the needs of athletes and the planet.

## 7. Conclusions

The application of superhydrophobic coatings in the waterproofing treatment of fitness equipment significantly enhances the durability, safety, and overall user experience. By reducing water friction and improving water resistance, these coatings help maintain the equipment’s integrity in various harsh environments, thereby increasing its lifespan and reducing maintenance costs. Additionally, superhydrophobic technology promotes better hygiene conditions in fitness settings by preventing water accumulation and limiting the growth of harmful bacteria. As technological advancements continue, the scope for applying superhydrophobic coatings in the fitness industry will only expand, driving further innovation and development.

Furthermore, superhydrophobic coatings show great potential in boosting the corrosion resistance of sports equipment. This technology not only helps gear withstand the effects of water and moisture but also ensures that equipment performs at its best over time. As a result, users can expect a more reliable, durable product that reduces maintenance needs while enhancing the overall sporting experience. The growing adoption of this technology will lead to a more sustainable and efficient approach to sports equipment production, benefiting both manufacturers and consumers.

Finally, the application of superhydrophobic coatings on sports equipment goes beyond water resistance, offering notable anti-icing capabilities. This feature is particularly beneficial for sports that are exposed to extreme conditions, where ice accumulation can hinder performance. By preventing the build-up of ice and maintaining the equipment’s functionality, superhydrophobic coatings contribute significantly to the enhancement in athletic performance. As the technology matures and becomes more widely adopted, it will play a crucial role in shaping the future of sports equipment, offering more protection and convenience to athletes across various disciplines.

## Figures and Tables

**Table 1:** Superhydrophobic theory summary.

Theory	ISS	SR	CH	DDB	SSD	STC
YM	✔					✔
WM		✔				✔
CBM		✔	✔		✔	
RACAH		✔	✔	✔	✔	
CLPM		✔	✔	✔	✔	

YM: Young’s Model. WM: Wenzel’s Model. CBM: Cassie–Baxter’s Model. RACAH: Rolling Angle and Contact Angle Hysteresis Theory. CLPM: Contact Line Pinning Model. ISS: ideal smooth surface. SR: surface roughness. CH: chemical heterogeneity. DDB: Dynamic droplet behavior. SSD: Superhydrophobic surface design. STC: Simple theory and calculation. ✔: This theory is consistent with this characteristic.

**Table 2:** Summary of the preparation method of superhydrophobic coatings.

Method	Advantage	Disadvantage	Materials Used
Template Method	Durable and mass production	High-cost and Complex process	Silane, Polymer
Sol-Gel Method	Strong hydrophobicity	High physical and chemical requirements	Silane, Nanoparticles
Coating Method	Fast speed and wide applicability	Low stability and durability	Silane, Fluoride
Physical Vapor Deposition	Low pollution, good abrasion resistance	High cost, slow sedimentation rate	Metal matrix material
Chemical Vapor Deposition	Adjustable deposit thickness	High cost, harsh environment	Silane, Metal oxide
Chemical Etching Method	Long service life, no adhesion problems	Uneven, highly polluting,	Acid, Alkaline, Fluoride
Laser Etching Method	Fast speed, no pollution, controllable	High energy consumption, high cost	Silane, Silicon dioxide
Plasma Technology	High efficiency, low energy consumption	Parameter optimization is difficult	Fluorine compound
